# Quercetin impairs *Streptococcus pneumoniae* biofilm formation by inhibiting sortase A activity

**DOI:** 10.1111/jcmm.13910

**Published:** 2018-10-18

**Authors:** Jianfeng Wang, Meng Song, Juan Pan, Xue Shen, Wentao Liu, Xueke Zhang, Hongen Li, Xuming Deng

**Affiliations:** ^1^ Department of Respiratory Medicine The First Hospital of Jilin University Changchun China; ^2^ Key Laboratory of Zoonosis Ministry of Education Institute of Zoonosis College of Veterinary Medicine Jilin University Changchun China; ^3^ Tianjin International Travel Healthcare Center Tianjin China; ^4^ Heilongjiang Veterinary Drug and Feed Super Vision Institute Haerbin China

**Keywords:** anti‐infection, biofilm, quercetin, sortase A, *Streptococcus pneumoniae*

## Abstract

Biofilm formation mediated by sortase A (srtA) is important for bacterial colonisation and resistance to antibiotics. Thus, the inhibitor of SrtA may represent a promising agent for bacterial infection. The structure of *Streptococcus pneumoniae* D39 srtA has been characterised by crystallisation. Site‐directed mutagenesis was used for the determination of the key residues for the activity of *S. pneumoniae* D39 srtA. An effective srtA inhibitor, quercetin, and its mechanism was further identified using srtA activity inhibition assay and molecular modelling. In this study, the crystal structure of *S. pneumoniae* D39 srtA has been solved and shown to contain a unique domain B. Additionally, its transpeptidase activity was evaluated in vitro. Based on the structure, we identified Cys207 as the catalytic residue, with His141 and Arg215 serving as binding sites for the peptide substrate. We found that quercetin can specifically compete with the natural substrate, leading to a significant decrease in the catalytic activity of this enzyme. In cells co‐cultured with this small molecule inhibitor, NanA cannot anchor to the cell wall effectively, and biofilm formation and biomass decrease significantly. Interestingly, when we supplemented cultures with sialic acid, a crucial signal for pneumococcal coloniation and the invasion of the host in the co‐culture system, biofilm loss did not occur. This result indicates that quercetin inhibits biofilm formation by affecting sialic acid production. In conclusion, the inhibition of pneumococcal srtA by the small molecule quercetin offers a novel strategy for pneumococcal preventative therapy.

## 1 INTRODUCTION

Pneumococcal infections, including pneumonia, otitis media, meningitis, and bacteremia, cause almost 2 million deaths annually.^1^ Biofilm formation plays an important role in bacterial survival and infection.^2^ For *Streptococcus pneumoniae*, biofilm formation is necessary for pneumococcal survival, early colonisation, and resistance to antimicrobial agents.^3^ Many factors are involved in biofilm formation; neuraminidase A (NanA) is one such factor.^4^

NanA, which is conserved in all strains and cleaves sugars from the mucosal surface, supports the release of sialic acid, which has been identified as a preventable signal for pneumococcal colonisation and invasion of the host by *S. pneumoniae*.^5^ Furthermore, NanA possesses an LPETG motif at the C terminus and therefore requires transpeptidation by *S. pneumoniae* sortase A (Spn‐srtA) for proper anchoring to the cell wall.^6^

Many surface proteins of Gram‐positive bacteria are anchored to the cell wall envelope by srtA, which recognises the conserved LPXTG motif, where X is any amino acid.^6^ The thiolate group of an essential active site Cys attacks the scissile Thr‐Gly bond, and two absolutely conserved residues, a His and an Arg, are located in the active site.^7^, ^8^, ^9^ Studies have shown that srtA from *Staphylococcus aureus*,* Bacillus anthracis*, and *Listeria monocytogenes* are directly related to bacterial adherence to host tissues.^6^

The *S. pneumoniae srtA* gene is widely expressed and highly conserved amongst isolated strains.^10^ Although *S. pneumoniae* TIGR4 has three additional putative sortase genes, *srtB*,* srtC* and *srtD*, these genes are absent in *S. pneumoniae* D39.^11^ Spn‐srtA has been shown to play a role in nasopharyngeal colonisation in chinchilla and adhesion to human pharyngeal cells in vitro.^11^, ^12^ Spn‐srtA is also important for pneumonia, bacteremia, and nasopharyngeal colonisation in mouse models, and it affects intraperitoneal immunity in mouse models. Evidence also suggests that Spn‐srtA is a promising candidate for a protein‐based pneumococcal vaccine.^10^, ^13^

Quercetin has been shown to inhibit the activity of SrtA from *Streptococcus mutans* and *Staphylococcal aureus*.^14^, ^15^ Here, we found that Spn‐srtA contains a domain B in addition to the domain A found in all sortases. Cys207 is the catalytic residue, and His141 and Arg215 are the binding sites for the peptide substrate. The small molecule quercetin efficiently reduced sortase activity, allowing NanA to be released into the culture. Herman‐Bausier et al^16^ showed that *S. aureus* fibronectin binding protein A mediates cell‐cell adhesion through low‐affinity homophilic bonds, and we observed a similar mechanism in *S. pneumoniae*: NanA self‐assembly assists pneumococcal aggregation and leads to biofilm formation. By preventing the normal anchoring of NanA, quercetin abrogated sialic acid production and bacterial aggregation and ultimately reduced pneumococcal biofilm formation. In summary, ablating Spn‐srtA activity and impairing pneumococcal biofilm formation seem to be a novel strategy for fighting pneumococcal infection.

## 2 MATERIALS AND METHODS

### 2.1 *Streptococcus pneumoniae* D39 srtA cloning, expression, and purification

The DNA sequence encoding Spn‐srtA residues Val‐82 to Tyr‐247 (srtA_∆N81_) was amplified from *S. pneumoniae* D39 genomic DNA using the primers 5′‐CTTAGGATCCATAAATCTTCATGCCAT‐3′ (forward) and 5′‐ATGTTCTCGAGTCTCAAAAAAATAATAAAAAG‐3′ (reverse). The amplified fragment was digested with *BamHI* and *XhoI* and cloned into a pGEX‐6P‐1 expression vector. The recombinant vector with an N‐terminal GST was transformed into *Escherichia coli* BL21 (DE3) cells for overexpression of the recombinant protein.

Cultures of BL21 (DE3) harbouring the srtA_∆N81_‐pGEX‐6P‐1 vector were grown at 37°C in a shaking incubator to an A_600_ of 0.6‐0.8 in Luria‐Bertani medium supplemented with ampicillin (100 mg/L). IPTG was then added to a final concentration of 0.5 mM, and the cultures were grown for a further 4 hours at 30°C prior to harvesting. Cell pellets were resuspended in phosphate buffered saline (PBS) and lysed by sonication. The lysed cells were centrifuged at 40 555 *g* at 4°C for 30 minutes.

The cell lysate was mixed with glutathione Sepharose beads (GE Healthcare, Uppsala, Sweden) that were pre‐equilibrated with PBS for 1 hour. The unbound proteins were washed away with PBS after loading onto the column. The eluted GST‐srtA_∆N81_ was mixed with PreScission protease at a ratio of 50:1 (w:w) in buffer A (25 mM HEPES, 100 mM NaCl, pH 7.0) and incubated at 4°C overnight to cleave the GST tag. Spn‐srtA_∆N81_ was further purified by ion exchange chromatography on a Resource Q column (GE Healthcare). Two peaks were obtained, and both were determined to contain srtA_∆N81_by SDS‐PAGE. Portions of both peaks were collected and concentrated before being loaded onto a Superdex 75 gel filtration column (GE Healthcare) pre‐equilibrated with buffer A. The fractions containing only Spn‐srtA_∆N81_ were pooled and concentrated to a final concentration of 15 mg/mL and stored at −20°C.

The Spn‐srtA_∆N81_ H141A, R215A, and C207A mutants were expressed and purified as described above for the wild‐type protein.

### 2.2 Crystallisation

SrtA_∆N81_ crystals were grown at 16°C using the hanging‐drop vapour diffusion method over a reservoir of 1.8 mol/L (NH_4_)_2_SO_4_ and 0.1 mol/L Bis‐Tris (pH 6.0). Crystals were grown to full size within 4 days.

### 2.3 Data collection and structure determination

Cryo‐cooling was carried out by soaking the crystals in a reservoir solution containing 20% (v/v) glycerol prior to flash‐cooling with liquid nitrogen. All of the diffraction data sets were collected at beam line BL1A of the Photon Factory synchrotron facility in Shanghai using a CCD detector, with a maximum resolution of ~3.3 Å. The space group of *S. pneumoniae*‐srtA_∆N81_ crystals is I4122, with 2 protein molecules per asymmetric unit. The initial phases were obtained with the molecular replacement method using the program Phaser v2.1,^17^ and the *S. aureus‐*srtA_∆N59_ crystal structure was used as the search model (Protein Data Bank [PDB] ID: 1T2P). Manual model building and refinement were performed with COOT^18^ and PHENIX^19^ using a rigid body, TLS parameters and individual B‐factor refinement. Solvent molecules were located based on stereochemically reasonable peaks in the σ_A_‐weighted 2Fo‐Fc difference electron density map. The quality of the final refined model was verified using the program PROCHECK.^20^ Final refinement statistics are summarised in Table 1. Structure figures were drawn using PyMOL.^21^

**Table 1:** Data collection and refinement statistics

Name	Spn‐srtA_∆N81_ (PDB ID: 5DV0)
Data collection	
Resolution (Å)	50.0‐3.30 (3.36‐3.30)
Space group	I4122
Cell dimensions	
*a* (Å)	117.6
*b* (Å)	117.6
*c* (Å)	101.9
Redundancy	12.9 (13.8)
Completeness (%)	99.9 (100.0)
*R*_merge_	0.045 (0.730)
*I*/σ (*I*)	48.9 (4.3)
Refinement	
Resolution (Å)	38.5‐3.3
No. reflections	5602
*R*_work_/*R*_free_	0.232/0.244
No. of non‐H atoms	
Protein	1330
B‐factors (Å^2^)	
Protein	69.4
Ramachandran statistics (%)	
Most favoured	92.17
Allowed	6.02
Outliers	1.81
R.M.S. deviations	
Bond lengths (Å)	0.003
Bond angles (°)	0.787

Values in parentheses are for the highest‐resolution shell.

^a^ *R*merge = ΣhklΣi|I(hkl)i − <I(hkl)>|/ΣhklΣiI(hkl)i.

^b^ *R*work = Σhkl|Fo(hkl) − Fc(hkl)|/ΣhklFo(hkl).

^c^ *R*free was calculated for a test set of reflections (5%) omitted from the refinement.

### 2.4 Site‐directed mutagenesis

Site‐directed mutagenesis of srtA_∆N81_ was performed to produce the H141A, R215A and C207A mutants using the QuickChange site‐directed mutagenesis kit (Stratagene, La Jolla, CA, USA). The primer pairs for these three mutations were as follows: srtA_∆N81_ H141A, forward: 5′‐GTCTAGCTAGTGCGCATATCTTTGG‐3′ reverse: 5′‐CCAAAGATATGCGCACTAGCTAGAC‐3′; R215A, forward: 5′‐CTGCTACAGAAGCGATTATTGTCAA‐3′, reverse: 5′‐TTGACAATAATCGCTTCTGTAGCAG‐3′; C207A, forward: 5′‐CATTAGTAACCGCGGAAGACCTTGC‐3′, reverse: 5′‐GCAAGGTCTTCCGCGGTTACTAATG‐3′.

### 2.5 Transpeptidation activity assay for Spn‐srtA_∆N81_, Spn‐srtA_∆N81_ H141A, Spn‐srtA_∆N81_R215A and Spn‐srtA_∆N81_C207A

The transpeptidation activities of the wild‐type and mutant proteins were determined by quantifying the increase in fluorescence intensity upon the cleavage of a synthetic peptide substrate. Reactions were performed in a 300 μl volume containing 5 mM Spn‐srtA_∆N81_ or Spn‐srtA_∆N81_H141A, Spn‐srtA_∆N81_R215A, or Spn‐srtA_∆N81_C207A in buffer B (50 mM Tris‐HCl, 5 mM CaCl_2_, and 150 mM NaCl, pH 7.5), and 10 mM Dabcyl‐QALPETGEE‐Edansor as the fluorescent peptide substrate. Reactions were carried out for 1 hour at 37°C and analysed fluorometrically (SpectraMAX Gemini XS; Molecular Devices Co., Sunnyvale, CA, U.S.A.) at 350 nm excitation and 510 nm emission.

### 2.6 Spn‐srtA_∆N81_ activity inhibition assay

Quercetin was prepared at different concentrations (0, 12.5, 25, 50, 100, and 200 μM) in DMSO and mixed with Spn‐srtA_∆N81_ for 15 minutes at 37°C. Samples were then analysed fluorometrically using the protocol described above. Blank samples (negative controls) contained all of the above except wild‐type Spn‐srtA.

### 2.7 Bacterial cultures

*Streptococcus pneumoniae* strain D39 was grown in Todd‐Hewitt broth (THB) + 2% yeast extract (THY media). Bacteria stored in glycerol at −80°C were thawed at room temperature and inoculated into fresh liquid THY medium. Bacteria were grown overnight to stationary phase at 37°C in a 5% CO_2_ incubator.

### 2.8 *Streptococcus pneumoniae* biofilm formation and biomass measurements

Mid‐log‐phase cell suspensions (1 × 10^8^ CFU/mL) were diluted 1:100 with fresh sterile THY media, and 1 mL aliquots were added in triplicate to the wells of a 24‐well flat‐bottom polystyrene microtitre plate with different concentrations of quercetin (0, 12.5, 25, 50, 100, and 200 μΜ). The plate was incubated statically for 15‐18 hours at 37°C, under 5% CO_2_. The OD_600_ was measured to determine the levels of bacterial growth. After incubation, the medium was aspirated, and the plates were gently washed three times with 1 mL of sterile PBS. Plates were then air‐dried and stained for 1 hour with 400 μL of 0.1% crystal violet (CV). Excess stain was decanted, and plates were washed three times with sterile distilled water. Plates were then allowed to dry, and the bound CV was dissolved in 200 μL of 95% ethanol. The OD_570_ was measured using a microplate reader. The data represent the average values of three replicates.

Biofilm biomass was quantified in terms of colony forming units (CFUs). *Streptococcus pneumoniae* biofilms were grown and washed as described above. Biofilm disaggregation was performed by gentle pipetting and slow vortexing. The biofilm bacteria were serially diluted, cultured on blood agar plates, and CFUs were counted.

### 2.9 Western blotting of pneumolysin

The bacteria were grown in the presence of different concentrations of quercetin (0, 12.5, 25, 50, and 100 μΜ) as described above. Bacterial pellets were lysed and heated. Equal amounts of protein were separated by 12% SDS‐PAGE and transferred to polyvinylidene fluoride membranes. The membranes were blocked, incubated with a monoclonal antibody against pneumolysin (1:1000; Abcam, Cambridge, UK), probed with HRP‐conjugated secondary anti‐mouse antibodies (1:2000; Proteintech, Chicago, IL, USA), and developed with ECL reagent (Thermo Scientific, Rockford, IL, USA). The protein bands were visualised using a Tanon‐4200 imager.

### 2.10 Supplementation of sialic acid in the quercetin and *S. pneumoniae* co‐culture system

*Streptococcus pneumoniae* D39 was grown in a 24‐well polystyrene microtitre plate with different concentrations of quercetin (0 or 100 μΜ) and sialic acid (0 or 3.2 mΜ) as described above. The biofilm was stained with 0.1% CV and quantified as described above.

### 2.11 Molecular modelling

**Figure 1:**
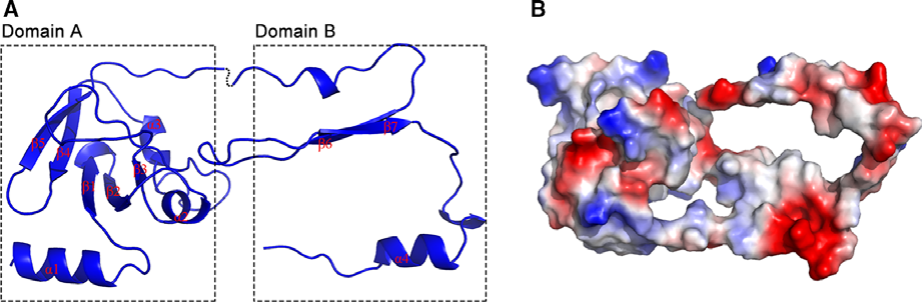
The structure and activity sites of Spn‐srtA_∆N81_. A, The overall structure of *Streptococcus pneumoniae* D39 srtA_∆N81_. B, Surface potential map of *S. pneumoniae* D39 srtA_∆N81_

In this work, the initial srtA structure was derived from the crystal structure (Figure 1). The starting structure of the ligand/srtA complex for the molecular dynamics (MD) simulation was obtained using AutoDock 4^22^ based on the standard docking procedure for a rigid protein and a flexible ligand. Then, the MD simulation was carried out for the complex using detailed computational biological processes described in previous reports.^23^, ^24^

### 2.12 Statistical analysis

Data were assessed using independent‐sample Student's *t* test (n ≥ 3). Analyses were performed using Graph Pad Prism (Graph Pad Software Inc., San Diego, CA, USA). Data are presented as mean ± SD. The significance levels of *P* < 0.05 and *P* < 0.01 between group means are indicated in the figures.

## 3 Results

### 3.1 The structure of *S. pneumoniae* D39 srtA

**Figure 2:**
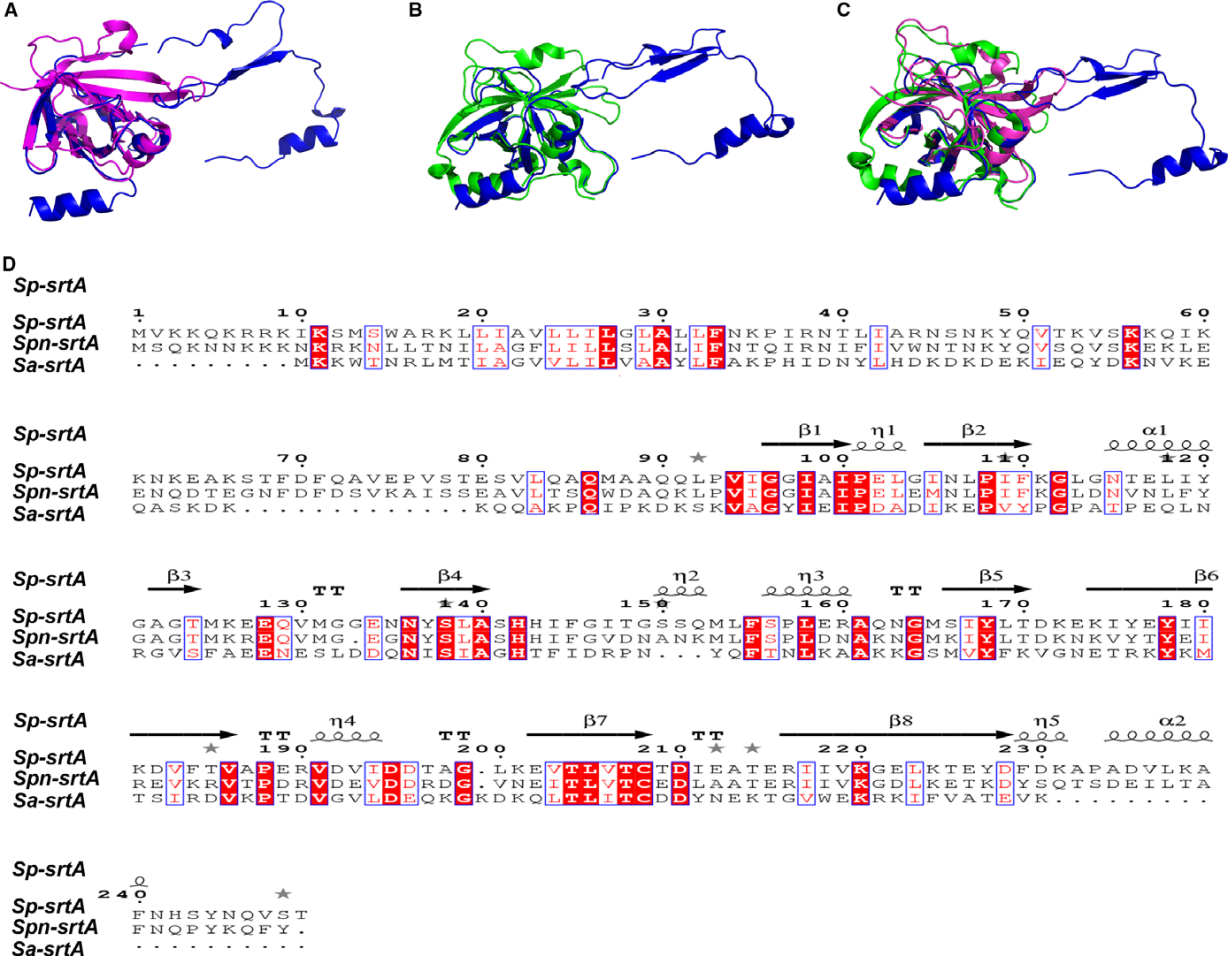
Structural and sequence comparisons between Sa‐srtA_∆N59_, Sp‐srtA_∆N81_, and Spn‐srtA_∆N81_. A, Structural comparison between Sa‐srtA_∆N59_ and Spn‐srtA_∆N81_. B, Structural comparison between Sp‐srtA_∆N81_ and Spn‐srtA_∆N81_. C, Structural comparison between Sa‐srtA_∆N59_, Sp‐srtA_∆N81_and Spn‐srtA_∆N81_. D, The amino acid sequences of Sa‐srtA_∆N59_, Sp‐srtA_∆N81_, and Spn‐srtA_∆N81_ were aligned using CLUSTALW2, and the alignment result was graphically displayed with the program ESPript. Secondary structural elements are indicated according to the structure of Sp‐srtA_∆N81_

The Spn‐srtA_∆N81_ structure contains 4 α‐helix and 7 β‐sheet folds and contains two domains (domain A and domain B) that are unique to sortases and whose structures have been previously described (Figure 1). Despite sharing 28% or even 56% sequence identity (Figure 2D), the overall structure of Spn‐srtA_∆N81_ is differed substantially from that of *S. aureus*‐srtA_∆N59_ (Sa‐srtA_∆N59_) and *S. pyogenes*‐srtA_∆N81_ (Sp‐srtA_∆N81_), which possess only domain A (Figure 2C). Sa‐srtA_∆N59_ and Sp‐srtA_∆N81_ can be overlaid on Spn‐srtA_∆N81_ with an RMSD of 1.8 Å (87 equivalent C_a_ atoms) (Figure 2A) and 23.6 Å (149 equivalent C_a_ atoms) (Figure 2B), respectively. The structure of Spn‐srtA_∆N81_ contains a huge hole formed by domain B.

### 3.2 Substrate recognition assay

**Figure 3:**
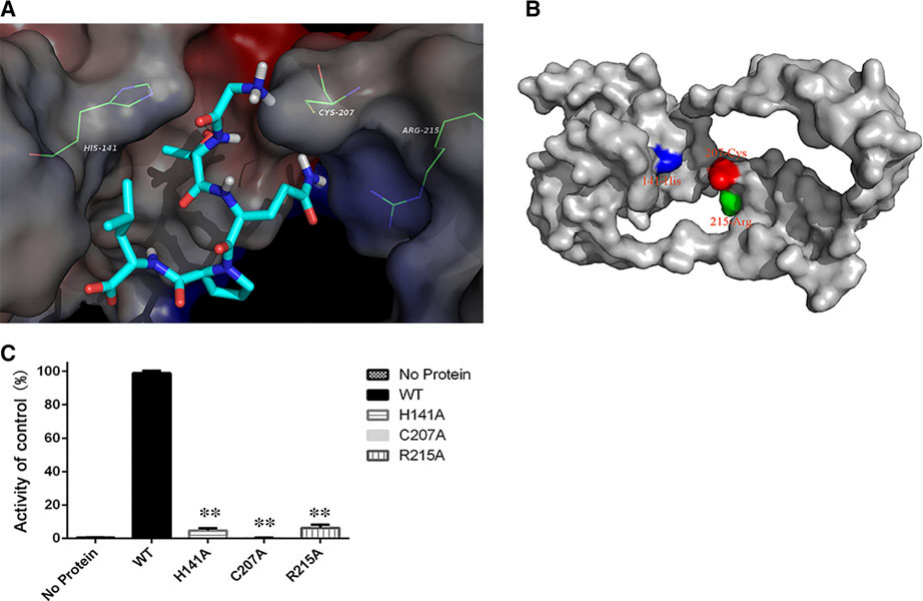
Substrate binding mode and the activity of Spn‐srtA_∆N81_ mutants. A, The binding mode of Spn‐srtA and the natural substrate peptide LPETG. B, The positions of His141, Cys207, and Arg215 in *Streptococcus pneumoniae* D39 srtA_∆N81_. C, Purified mutant protein used to determine Spn‐srtA_∆N81_ activity compared to the wild‐type enzyme (WT). Error bars are ± SD. n = 5. ** indicates *P* < 0.01 compared with the wild‐type srtA_∆N81_

To gain further insights into the interaction between Spn‐srtA_∆N81_ and its substrates and to analyse its cleavage specificity, an oligopeptide substrate was modelled in the substrate binding site in silico. The structure of Sa‐srtA in complex with its LPETG substrate (PDB entry: 1T2W) was used as the basis for the initial modelling. As shown in Figure 3A, the results indicated that the two “hand” residues His141 and Arg215 catch the LPETG peptide, which is then cleaved by the active site residue Cys207.

### 3.3 His141 and Arg215 are the binding sites and Cys207 is the catalytic residue for the peptide substrate

Previous studies of Sa‐srtA and Sp‐srtA identified Cys208, His142, and Arg216 as the key catalytic residues (Figure 3B). We found that although the Spn‐srtA structure differs from those of Sa‐srtA and Sp‐srtA, the active residues of Spn‐srtA are also His141, Cys207, and Arg215. Spn‐srtA loses its transpeptidation activity if these three amino acids are mutated (Figure 3C).

### 3.4 Quercetin inhibits *S. pneumoniae* srtA activity

**Figure 4:**
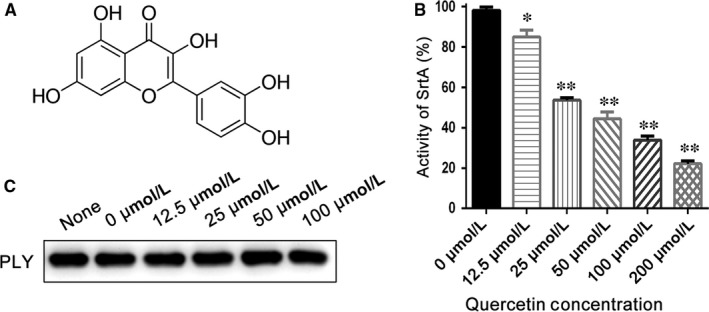
The inhibitory effect of quercetin on Spn‐srtA and western blotting of pneumolysin expression. A, The structure of quercetin. B, Inhibition of Spn‐SrtA activity in the presence of various concentrations of quercetin (0‐100 μM). C, Western blot analysis of pneumolysin expression in *S. pneumonia* D39 cultured with different concentrations of quercetin (0‐100 μM). The samples treated with 0 μM quercetin were exposed to an equal volume of DMSO. Error bars are ± SD. n = 5. * indicates *P* < 0.05 and ** indicates *P* < 0.01 compared with the quercetin‐free sample

The activity of purified Spn‐srtA_∆N81_ was measured in the presence of different concentrations of quercetin (Figure 4A) (0, 12.5, 25, 50, 100, and 200 μΜ), and quercetin was discovered to significantly inhibit Spn‐srtA_∆N81_ (*P* < 0.01) catalytic activity in a dose‐dependent manner (Figure 4B).

### 3.5 Quercetin impairs pneumococcal biofilm formation and reduces biomass

**Figure 5:**
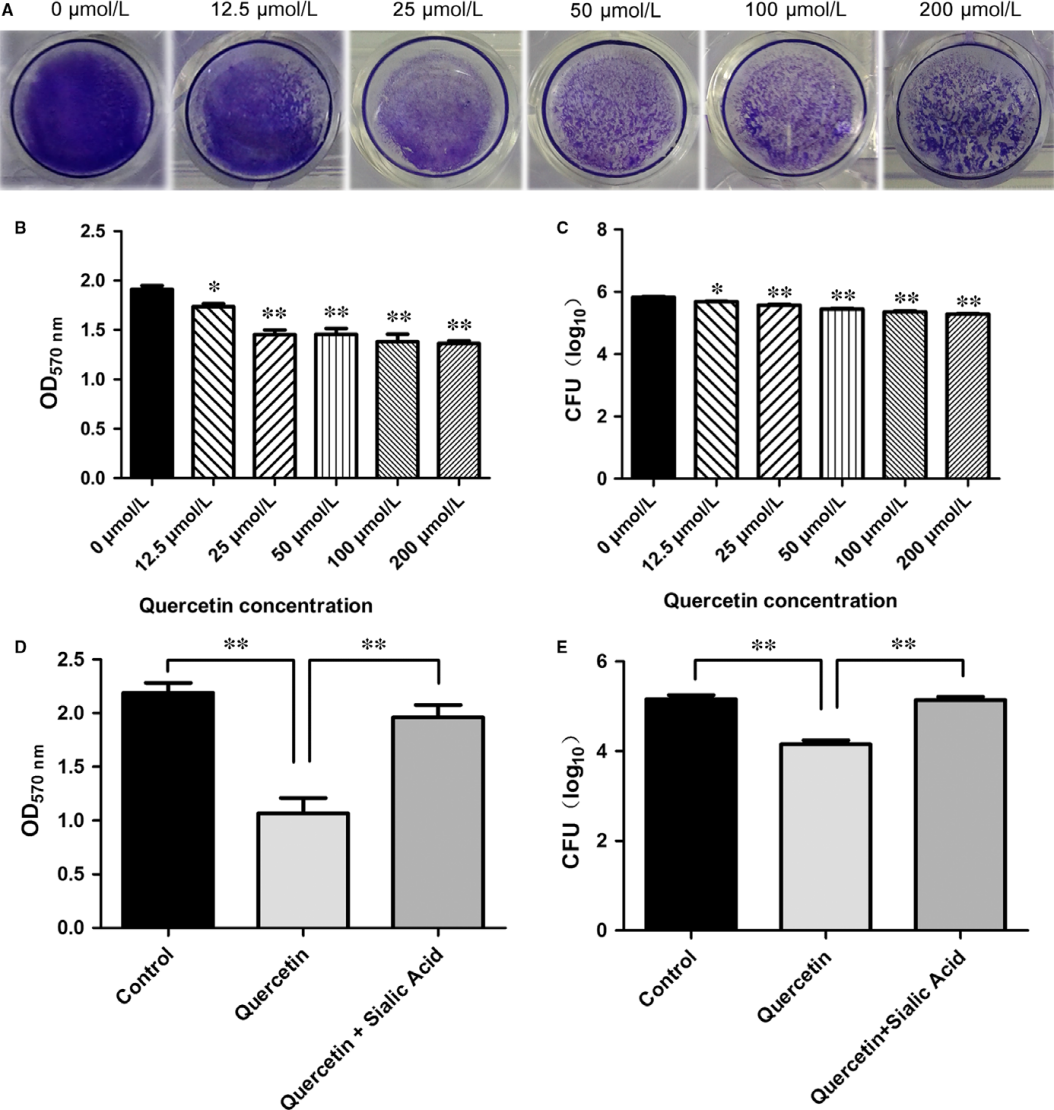
Effect of quercetin on *Streptococcus pneumoniae* biofilm formation and biomass. A, Photograph of crystal violet‐stained biofilms grown with different concentrations of quercetin (0‐200 μM). B, Quantification of biofilm biomass via crystal‐violet staining. C, CFU counts of biofilms with different concentrations of quercetin (0‐200 μM). D and E, The quercetin‐impaired pneumococcal biofilms recovered after the addition of excess sialic acid. The samples treated with 0 μM quercetin or control samples were exposed to equal volume of DMSO. Error bars are ± SD. n = 5. * indicates *P* < 0.05 and ** indicates *P* < 0.01 compared with the quercetin‐free sample

Parker and coworkers demonstrated that NanA is involved in biofilm formation and showed that a NanA mutant has significantly reduced activity compared to the wild‐type in *S. pneumoniae* D39.^4^ Thus, we examined whether a small molecule inhibitor could prevent pneumococcal biofilm formation. Indeed, pneumococcal biofilm formation was reduced with increasing quercetin concentration. The reduction in biofilm formation was indicated by the reduced intensity of CV staining (Figure 5A). Biofilm formation with 12.5 μM quercetin was significantly (*P* < 0.01) reduced compared to biofilm formation without quercetin (Figure 5B). The biofilm biomass was measured by counting CFU. Compared to biofilm growth in the absence of quercetin, biofilms grown in the presence of quercetin concentrations ≥12.5 μM displayed significantly reduced biomass (*P* < 0.05; Figure 5C). Additionally, Shak et al previously revealed an unrecognised role for pneumolysin in biofilm formation.^25^ We further tested the effect of quercetin on pneumolysin expression via western blotting and found that quercetin had no effect on pneumolysin expression (Figure 4C). Thus, the loss of pneumococcal biofilm formation caused by quercetin was exclusively due to the inhibition of Spn‐srtA activity.

### 3.6 Sialic acid reverses quercetin‐mediated inhibition for the pneumococcal biofilm formation

Trappetti et al found that sialic acid could reverse the inhibition of biofilm formation caused by mutating NanA.^26^ Our results revealed that sialic acid treatment similarly restored biofilm formation following the disruption of pneumococcal srtA activity. We added 3.2 mM sialic acid and 50 μM quercetin to a bacterial co‐culture system and found that biofilm formation by *S. pneumoniae* D39 recovered to the same level as that in the positive control (Figure 5D). In agreement with this result, the inhibitory effect of quercetin (50 μM) on biofilm growth was reduced in the presence of 3.2 mM sialic acid (Figure 5E).

The biofilm biomass was measured by counting CFU. Compared to biofilm growth in the absence of quercetin, biofilms grown in the presence of quercetin concentrations ≥12.5 μM displayed significantly reduced biomass.

### 3.7 Molecular modelling

**Figure 6:**
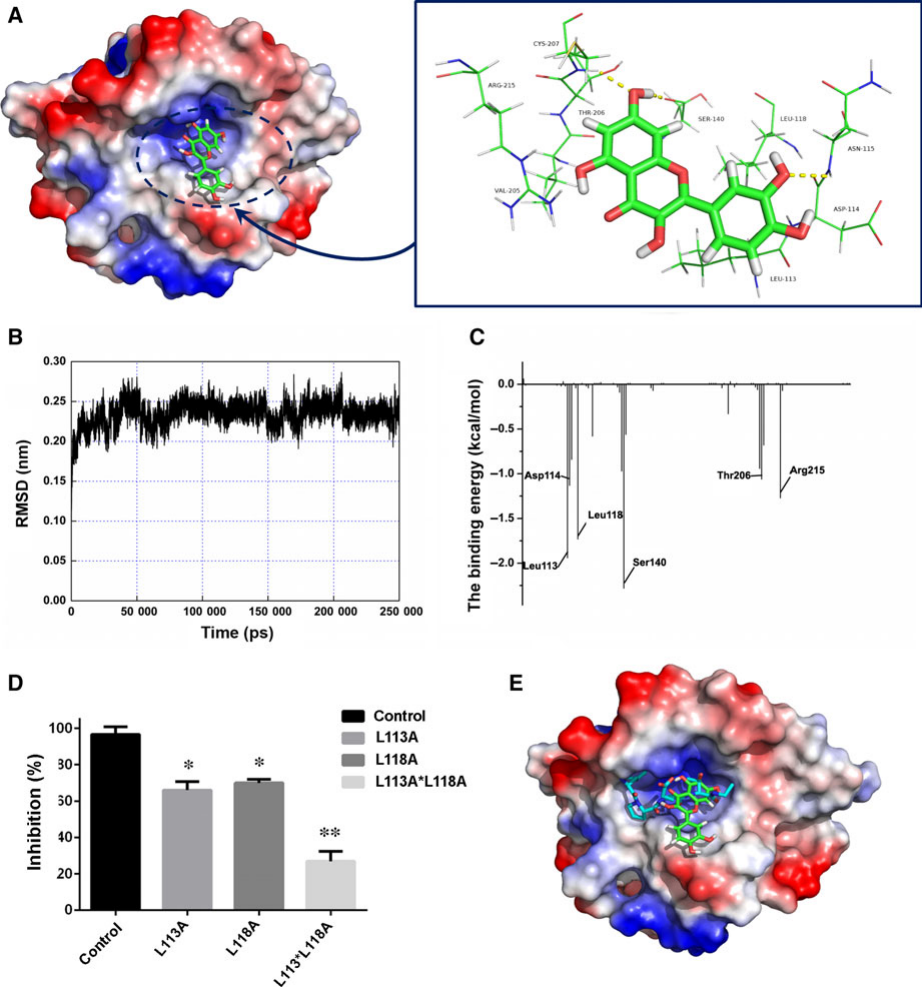
The mechanism of srtA inhibition by quercetin. A, The RMSDs displayed by the backbone atoms of the protein during the MD simulation of srtA with quercetin. B, RMSF of the residue positions with respect to their initial position in the free protein and in complex with quercetin. C, The decomposition of the binding free energy on a per‐residue basis in the binding sites of the srtA and quercetin complex. D, Mutating either L113A or L118A inhibits Spn‐srtA activity reduced the inhibitory activity of quercetin towards Spn‐srtA. The control samples were exposed to equal volume of DMSO. E, The model showing quercetin occupying the substrate channel, which causes steric hindrance between the substrate and Spn‐srtA. Error bars are ± SD. n = 5. * indicates *P* < 0.05 and ** indicates *P* < 0.01 compared to the control (wild‐type srtA_∆N81_)

In this work, we initially obtained a crystal structure of the srtA dimer. However, the biologically active form of srtA is a monomer in vivo. To explore the structural features of srtA, MD simulations were performed using the crystal structure of srtA as the initial structure. During a 200‐ns MD simulation, the 3‐D structure of monomeric srtA was first equilibrated with the solute (Figure 6A‐C). This was further used to model the molecular docking of quercetin. The data showed that mutating either Leu113 or Leu118 (Figure 6D) significantly reduced the inhibitory activity of quercetin towards Spn‐srtA, indicating that the inhibitor occupies the channel where the substrate binds to the protein, causing steric hindrance between the substrate and Spn‐srtA (Figure 6E).

## 4 Discussion

Bacterial pathogens have evolved numerous defence mechanisms against antibiotics that have traditionally targeted key components of the bacterial growth cycle and conferred evolutionary pressure on targeted bacteria. In 1985, Liu and Tomasz identified five clinical isolates of *S. pneumoniae* in South Africa that displayed tolerance to penicillin.^27^ In addition, Vancomycin‐tolerant *S. pneumoniae* has recently been identified in several countries.^28^ There are now fewer effective antibiotics that are available for fighting the pneumococcal infection.

Fortunately, the emergence of new anti‐infective strategies that target the bacterial infection process has begun to ease this problem to some extent. For example, baicalin is able to protect mice from *S. aureus* pneumonia by inhibiting the cytolytic activity of α‐hemolysin,^29^ and fisetin has also proven effective in attenuating *L. monocytogenes* infection by targeting listeriolysin O.^30^ The sortase mutants for *S. aureus* and *Streptococcus suis* were previously identified to interfere with the display of surface proteins and affects pathogenesis in animals.^31^, ^32^ Therefore, srtA has received considerable attention as a target for fighting bacterial infection. Many srtA structures have been elucidated,^33^, ^34^, ^35^ and molecular inhibitors have been found or designed for Gram‐positive infections.^36^, ^37^, ^38^

**Figure 7:**
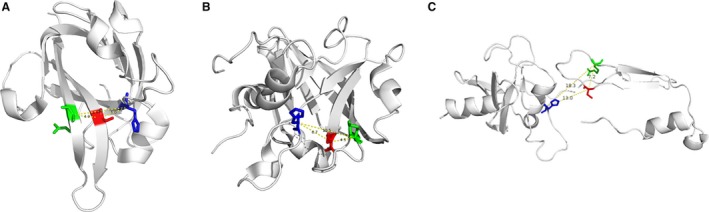
The distance between His, Cys, and Arg in Sa‐srtA_∆N59_, Sp‐srtA_∆N81_, and Spn‐srtA_∆N81_. A, The distance between His, Cys, and Arg in Sa‐srtA_∆N59_. B, The distance between His, Cys, and Arg in Sp‐srtA_∆N81_. C, The distance between His, Cys, and Arg in Spn‐srtA_∆N81_. Blue is His, red is Cys and green is Arg

In this study, we present the crystal structure of Spn‐srtA composed of a domain A and a domain B, which differs from the structure of srtA in *S. aureus* and *S. pyogenes*. However, consistent with other reports srtA, His141, Cys207, and Arg215 are still the active residues for Spn‐srtA activity. Mutation of any of these three amino acids results in significant loss of srtA transpeptidation activity. Additionally, our findings provide a probable explanation regarding why *S. pneumoniae* D39 only expresses srtA, whereas srtA, sortase B, sortase C, and sortase D coexist in *S. aureus* and *S. pyogenes*. The three active sites of Spn‐srtA, His141, Cys207, and Arg215, are located on loops, which are more flexible than α‐helixes and β‐sheets. Therefore, the His‐Cys, Cys‐Arg, and Arg‐His distances are 13 Å, 7.2 Å and 18.3 Å, respectively, which are further distances than those in Sa‐srtA (5.0 Å, 4.9 Å, and 9.5 Å) and in Sp‐srtA (6.7 Å, 4.7 Å, and 10.5 Å) (Figure 7). This observation implies that these three residues are very flexible and can accommodate more space, which may enable the processing of *S. pneumoniae* TIGR4 sortase B, sortase C and sortase D or other peptide substrates.

Using a transpeptidation activity assay, quercetin was identified as an effective inhibitor of *S. pneumoniae* srtA by inhibiting Spn‐srtA transpeptidase activity. Furthermore, quercetin treatment reduced pneumococcal biofilm formation. Finally, when sialic acid is provided, quercetin is no longer able to inhibit pneumococcal biofilm formation. These results indicate that the small molecule quercetin impairs pneumococcal biofilm formation by directly blocking the anchoring of pneumococcal NanA and indirectly reducing sialic acid production. NanA and biofilm formation are required for pneumococcal colonisation and infection of the upper and lower respiratory tract, respectively. A NanA mutant is cleared from the nasopharynx, trachea, and lungs within 12 hours post‐infection and is unable to persist in the blood beyond 48 hours post‐infection in vivo.^39^ Biofilm formation facilitates the ability of *S. pneumoniae* to evade complement immunity and phagocytosis by diverting alternative complement pathway activation through a PspC‐mediated mechanism.^40^ Additionally, Trappetti et al proved that sialic acid is a pivotal signal for the pneumococcal biofilm formation, colonisation, and host invasion.^5^

Molecular modelling simulation analysis has revealed the engagement of quercetin with the channel of Spn‐srtA, where the substrate binds to the protein, hinders the recognition of Spn‐srtA and its substrate. Resides Leu113 or Leu118 in Spn‐srtA are critical for such engagement, as validated by mutational analysis. Our results provide evidence that quercetin is a promising inhibitor for Spn‐srtA transpeptidase activity by directly occupying the substrate binding region of this enzyme protein. Thus, quercetin is a potent small‐molecule antibiotic that can fight the pneumococcal infection by specially targeting srtA.

## CONFLICT OF INTEREST

The authors declare no competing financial interests.
